# Supplementary material for: Removal of Cu(II) Contamination from Aqueous Solution by Ethylenediamine@β-Zeolite Composite
Source: Molecules. 2021 Feb 12;26(4):978. doi: 10.3390/molecules26040978 (PMC7917737; doi:10.3390/molecules26040978)
Supplement: Supplementary file 1 [file molecules-26-00978-s001.pdf]

Supporting Information

Figure S1. Leaching efficiency of EDA@β-zeolite.

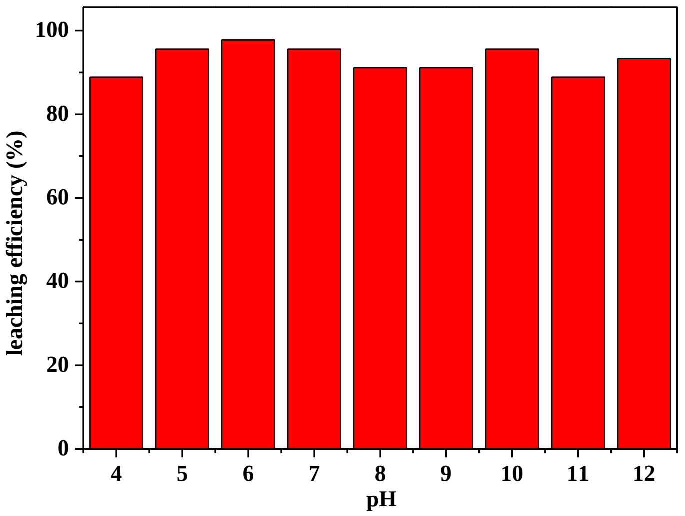

Table S1. Comparison of the Langmuir, Freundlich and D-R adsorption constants of Cu(II) onto β-zeolite and EDA@β-zeolite.

T (K)	pH	Sample	Langmuir model				Freundlich model			D-R model			
			K_a (L/mol)	q_{max} (mol/g)	R_L	R^2	K_F (mmol/g)	n	R^2	K (mol^2/kg^2)	q_m (mol/g)	E (kJ/mol)	R^2
298	5.10	β-zeolite	8080.28	5.94×10^{-5}	0.55	0.9896	0.06	2.60	0.9025	8.14×10^{-7}	2.26×10^{-4}	247.89	0.9271
		EDA@β-zeolite	1499.07	7.50×10^{-5}	0.87	0.9982	0.05	3.54	0.8908	5.46×10^{-7}	1.93×10^{-4}	302.59	0.9354
	6.10	β-zeolite	1632.49	1.25×10^{-4}	0.86	0.9960	0.09	2.36	0.8656	8.19×10^{-6}	5.31×10^{-4}	247.10	0.9072
		EDA@β-zeolite	998.84	1.66×10^{-4}	0.91	0.9985	0.09	2.46	0.9129	5.93×10^{-6}	4.25×10^{-4}	290.26	0.9375
318	5.10	β-zeolite	5648.25	7.20×10^{-5}	0.64	0.9967	0.04	3.56	0.9562	5.61×10^{-7}	1.80×10^{-4}	298.66	0.9717
		EDA@β-zeolite	231.20	9.45×10^{-5}	0.98	0.9928	0.03	6.03	0.8374	3.14×10^{-7}	1.72×10^{-4}	399.08	0.8852
	6.10	β-zeolite	1346.58	1.57×10^{-4}	0.88	0.9994	0.08	2.77	0.9312	7.13×10^{-6}	5.13×10^{-4}	264.89	0.9594
		EDA@β-zeolite	403.00	2.14×10^{-4}	0.96	0.9986	0.06	4.44	0.9290	4.45×10^{-6}	4.41×10^{-4}	335.27	0.9567
